# A bioinformatics investigation into the pharmacological mechanisms of the effect of Fufang Danshen on pain based on methodologies of network pharmacology

**DOI:** 10.1038/s41598-019-40694-4

**Published:** 2019-04-11

**Authors:** Yantao Sun, Jie Yang

**Affiliations:** School of life sciences, Nanjing University, Nanjing, 210023 China

## Abstract

Fufang Danshen (FFDS), a Chinese medicine formula widely used in the clinic, has proven therapeutic effects on pain relief. However, the mechanisms of these effects have not been elucidated. Here, we performed a systematic analysis to discover the mechanisms of FFDS in attenuating pain to gain a better understanding of FFDS in the treatment of other diseases accompanied by pain. Relevance analysis showed that Salvia miltiorrhizae was the best studied herb in FFDS. Most compounds in FFDS have good bioavailability, and we collected 223 targets for 35 compounds in FFDS. These targets were significantly enriched in many pathways related to pain and can be classified as signal transduction, endocrine system, nervous system and lipid metabolism. We compared Salvia miltiorrhizae and Panax notoginseng and found that they can significantly affect different pathways. Moreover, ten pain disease proteins and 45 therapeutic targets can be directly targeted by FFDS. All 45 therapeutic targets have direct or indirect connections with pain disease proteins. Forty-six pain disease proteins can be indirectly affected by FFDS, especially through heat shock cognate 71 kDa protein (HSPA8) and transcription factor AP-1 (JUN). A total of 109 targets of FFDS were identified as significant targets.

## Introduction

Pain, a major symptom related to cancer, inflammation and other diseases, has been defined as an unpleasant sensory and emotional experience associated with actual or potential tissue damage or described in terms of such damage by the International Association for the Study of Pain (IASP)^1^. Pain is not always good for us. In a normal state, pain may help us avoid injury, but in a pathological state, it evolves from a symptom indicating tissue damage to a disease itself ^2^. The mechanisms accounting for pain have not yet been fully elucidated. The discovery of neurons and their roles in pain^3^ invalidated many theories related to pain. Currently, the specificity or labeled line theory and gate control theory are the most controversial topics. According to different characteristics, pain can be classified into various types, such as acute pain, chronic pain, inflammatory pain, and neuropathic pain. Clinical pain is a serious public health issue. As the primary drugs, opioids and nonsteroidal anti-inflammatory drugs (NSAIDS) are the most widely used in the treatment of pain. However, these drugs have many severe adverse effects that have often been observed in a large number of patients. Common side effects of opioids include constipation, nausea, vomiting, respiratory depression, and urinary retention. Common side effects of NASIDS include injury to the gastrointestinal tract, liver and kidney dysfunction, and hematological system damage. All of these factors have necessitated the development of alternative analgesics^4^. Although many new analgesics have been introduced to the clinic for the treatment of pain in the past decades, we cannot deny the lack of real breakthrough drugs in clinical pain control^5^. Improvements have been made in our understanding of pain mechanisms, and many therapeutic targets and disease proteins related to pain have been found^6^. However, there may be more undiscovered successful drugs for treating various types of pain.

Traditional Chinese medicine (TCM) is an important part of world medicine. Despite its unknown molecular mechanisms, the therapeutic effects of TCM on curing diseases are recognized by hundreds and thousands of people. TCM is an important complementary and alternative medicine accepted by 183 countries and regions worldwide. The most famous theory of TCM is the balance-regulation theory, which emphasizes the integrity of the human body as well as the interaction between human individuals and their environment^7^. Scientists have studied herbal medicines and found that more than 800 types of TCM are effective in relieving pain^8^. Fufang Danshen (FFDS), recorded in Chinese Pharmacopoeia (2015), has been used clinically to treat coronary arteriosclerosis, angina pectoris, hyperlipemia and Alzheimer’s disease in China and is also available as a dietary supplement or drug in other countries^9^. FFDS comprises three herbs, including Salvia miltiorrhizae (Danshen) as a Jun drug (monarch drug), Panax notoginseng (Sanqi) as a Chen drug (ministerial drug), and Borneolum (Bing Pian) as Zuo and Shi drugs (adjuvant drug and messenger drug). In TCM, herbal formulas are organized based on the rule of “Jun-Chen-Zuo-Shi” to synergize therapeutic effects and integrally minimize adverse effects^10,11^. Many studies have proven that FFDS has many biological functions, including relieving pain, promoting blood circulation, improving reduced blood flow, reducing blood lipids, protecting blood vessels and myocardium, and improving heart function^12–14^. Herbs in this formula were recently found to affect other diseases, such as cancer, and osteoporosis^15,16^. Although many studies have demonstrated the significant therapeutic effects of FFDS on attenuating neuropathic pain, cancer pain, osteoarthritis pain, migraine and angina pectoris, few studies have been conducted to uncover the mechanisms. As pain is a common, severe symptom related to diseases that FFDS can cure, uncovering the mechanisms of this formula in treating pain will provide a better understanding of FFDS in the treatment of those diseases.

Network pharmacology is an approach to drug design that encompasses systems biology, network analysis, connectivity, redundancy and pleiotropy^17^. Network pharmacology is recognized as a new strategy and powerful tool for the exploration of drug targets and the identification of potentially active ingredients in TCM research^18,19^. In the present study, we conducted a bioinformatics investigation to elucidate the multilevel mechanisms of FFDS in attenuating pain. This investigation includes the following main steps: (1) assess the relevance of FFDS and its herbs with pain; (2) drug-likeness and bioavailability analysis of compounds in FFDS; (3) collection of targets for FFDS; and (4) analysis of the potential pharmacological mechanisms of FFDS in the treatment of pain. Compared with other TCM network pharmacology approaches, such as Li’s^20^, our approach focused on uncovering potential pathways and processes that a herbal formula affects to cure a disease. Similar steps include preparing data (compounds in a formula, target collection or prediction for a formula, disease proteins and therapeutic targets or proteins involved in disease-related pathways, and protein-protein interaction (PPI) data), analyzing networks and discovering the potential pathways or processes that the formula affects to cure a specific disease. Most TCM network pharmacology studies or informatics investigations focus on uncovering the mechanisms of action of an entire formula and ignore the discovery of active compounds in the formula (with the exception of Li^21^, who has introduced a novel method to predict active compounds in a formula), which will hinder the modernization of TCM. In our investigation, we used the “drug-target” principle and “essential protein” theory, which suggests that “a highly connected protein is more important” to uncover potentially active compounds for the treatment of pain. Our results successfully uncovered some active compounds that have experimentally proven antinociceptive effects. In addition, we analyzed disease proteins and therapeutic targets separately since they are different concepts, while most studies analyze them as a whole. Disease proteins are products of disease genes, which are the main factors that can cause diseases, but therapeutic targets are important factors that can cure diseases. We also evaluated therapeutic targets based on their degrees in the drug-target network, and we suggested that therapeutic targets with higher degrees were more likely or more efficient therapeutic targets for a specific disease. In addition, we discussed the relevance of FFDS and its herbs with pain based on text mining. The discussion covers the extent to which FFDS and its herbs have been associated with pain and which herb is more efficient for the treatment of pain. Based on these TCM network pharmacology approaches, we will have a clearer, further understanding of the pharmacological mechanisms of FFDS in attenuating pain.

## Results

### FFDS, its herbs and pain

As shown in Table 1, Salvia miltiorrhizae (43 papers) is the best studied herb in FFDS due to its importance in TCM, followed by Panax notoginseng (13 papers). Pain is a major, common symptom related to various diseases; thus, the volume of research on pain is extensive (599207). The number of papers relating FFDS or its herbs to pain is very small, and the P-value for FFDS and Panax notoginseng was nonsignificant, which may be because western medicine is more efficient and convenient than TCM in the treatment of pain. Additionally, TCM is preferred for use in complex diseases, such as cancer and Alzheimer’s disease. The study of TCM in the treatment of pain can provide a better understanding of its efficacy in the treatment of other diseases accompanied by pain and aid in the development of new analgesics from TCM.

**Table 1:** Relevance between FFDS, its herbs and pain.

Herb	Total	Relevant to diseases	Relevant to pain	Ratio	P-value
Fufang Danshen	38	15	1	6.67%	0.2858
Salvia miltiorrhizae	2296	1105	43	3.89%	0.0049
Panax notoginseng	1031	410	13	3.17%	0.1291
Borneolum	25	16	3	18.75%	0.0004

The ratio is the volume of papers relevant to pain/the volume of papers relevant to diseases.

### Drug-likeness and bioavailability analysis for compounds in FFDS

Ultimately, we obtained 123 compounds for Salvia miltiorrhizae (Danshen), 78 compounds for Panax notoginseng (Sanqi), and 20 compounds for Borneolum (Bing Pian). Dauricine and gamma-Sitosterol are shared by Salvia miltiorrhizae and Panax notoginseng; Bata-Caryophyllene and Elemicin are shared by Panax notoginseng and Borneolum. By contrast, the other compounds belong to only one of the three herbs. In total, 217 compounds were obtained for FFDS. The detailed information is listed in Table S1. The statistics for the six main properties of all compounds in FFDS are shown in Table 2. As shown, the mean and median values of properties of compounds in FFDS conform to “the rule of five”, and most (173/217) of the ingredients in FFDS conform to drug-likeness rules. A bioavailability score was also obtained to evaluate the bioavailability of ingredients in FFDS. The result showed that 75.6% of ingredients have good bioavailability. Conforming to the predictions, the poor bioavailability of ginsenosides and salvianolic acids has been proven.

**Table 2:** Statistics of the six main properties of compounds in FFDS.

Descriptors	Max value	Min value	Mean value	Median value
TPSA	447.21	0	105.52	67.51
Num. H-bond acceptors	27	0	6.28	4
Num. H-bond donors	18	0	3.29	1
Num. rotatable bonds	21	0	4.82	2
MLOGP	6.92	−6.15	1.46	1.48
Molecular weight (MW)	1269.46	44.1	402.74	311.33

### FFDS targets and their functions

A total of 223 targets were retained for 35 compounds, with 99 targets belonging to Salvia miltiorrhizae, 143 targets to Panax notoginseng and 17 targets to Borneolum. Among the 35 compounds, 18 compounds belong to Salvia miltiorrhizae, 17 belong to Panax notoginseng, and 2 belong to Borneolum. Based on these two perspectives, we concluded that the rule of “Jun-Chen-Zuo-Shi” is reasonable to organize herbs in a formula. Detailed information is available in Table S2. Salvia miltiorrhizae and Panax notoginseng shared 24 targets; Salvia miltiorrhizae and Borneolum shared 7 targets; Panax notoginseng and Borneolum shared 8 targets; and cytochrome P450 3A4 (CYP3A4), high mobility group protein B1 (HMGB1), and caspase-3 (CASP3) belong to the three herbs. Seventy-one targets belong to only Salvia miltiorrhizae, and these targets were significantly enriched in steroid hormone biosynthesis, HIF-1 signaling pathway, Jak-STAT signaling pathway and PI3K-Akt signaling pathway. These four pathways have relationships with pain and may constitute the mechanisms of Salvia miltiorrhizae in the treatment of pain. For example, steroids can affect the nervous system and are of particular interest in the modulation of pain^22,23^; molecules targeting the Jak-STAT signaling cascade are successful even though the specific contribution of this pathway in the modulation of pain is unknown^24^. Additionally, Jak-STAT and PI3K-Akt are important microglial intracellular signaling cascades that are essential for neuropathic pain development and maintenance^25^. A total of 114 targets belong to only Panax notoginseng, and they were significantly enriched in 13 pathways related to pain, including arachidonic acid metabolism, adipocytokine signaling pathway, tumor necrosis factor (TNF) signaling pathway, serotonergic synapse, inflammatory mediator regulation of TRP channels, Toll-like receptor signaling pathway, linoleic acid metabolism, oxytocin signaling pathway, NF-kappa B signaling pathway, MAPK signaling pathway, GnRH signaling pathway, mTOR signaling pathway and vascular endothelial growth factor (VEGF) signaling pathway, which were ranked by P-values. Leptin, an adipocytokine, plays an important role in nociceptive behavior induced by nerve injury^26^. Modulators of the arachidonic acid cascade have been the focus of research on treatments for inflammation and pain for several decades, and the design and development of multitarget inhibitors of this pathway that exhibit improved efficacy and less undesired side effects is a new paradigm^27^. The targets belonging to only Salvia miltiorrhizae or Panax notoginseng were also involved in pathways significantly enriched by targets belonging to the opposite herb only. For example, the targets of Panax notoginseng were involved in the HIF-1 signaling pathway and PI3K-Akt signaling pathway, which were significantly enriched by targets of Salvia miltiorrhizae. Additionally, targets of Salvia miltiorrhizae were involved in the TNF signaling pathway and NF-kappa B signaling pathway, which were significantly enriched by targets of Panax notoginseng. Five targets belong to only Borneolum, while 12 targets are shared with Salvia miltiorrhizae or Panax notoginseng. Five unique targets, including UDP-glucuronosyltransferase 2B10 (UGT2B10), UDP-glucuronosyltransferase 2B11 (UGT2B11), Nuclear factor erythroid 2-related factor 2 (NFE2L2), NAD(P)H dehydrogenase [quinone] 1 (NQO1) and Prostacyclin receptor (PTGIR), may explain why Borneolum is necessary for the formula. UGT2B10, UGT2B11 and NQO1 play important roles in the elimination of toxic materials. NFE2L2 is a transcription activator that binds to antioxidant response elements (AREs) in the promoter regions of target genes and is important for the coordinated upregulation of genes in response to oxidative stress. FFDS has further potential to exert therapeutic effects through targets with at least two herbs or compounds; thus, we retained 51 targets with at least two herbs or compounds in Table S3. Thirteen targets can interact with at least three compounds, and CASP3 is targeted by the most compounds (7), followed by CYP3A4 (4). Of note, Salvia miltiorrhizae and Panax notoginseng possess the most targets and most of the same targets, indicating that they are major herbs in FFDS. However, Borneolum is also essential for its unique targets.

Subsequently, we performed enrichment analysis for 223 FFDS targets, and pathways with P-value <= 0.01 and Fold Enrichment >= 1.5 were retained to find their relationships with pain. A total of 26 pathways may have relationships with pain. Among the 26 pathways, fourteen pathways with Bonferroni <= 0.01 were retained in Table 3. The other 12 pathways include the ErbB signaling pathway, prolactin signaling pathway, oxytocin signaling pathway, insulin signaling pathway, sphingolipid signaling pathway, neurotrophin signaling pathway, GnRH signaling pathway, cholinergic synapse, Jak-STAT signaling pathway, inflammatory mediator regulation of TRP channels, estrogen signaling pathway and AMPK signaling pathway. These pathways were also significantly enriched by targets with at least two compounds except for the adipocytokine signaling pathway, NF-kappa B signaling pathway, ErbB signaling pathway, GnRH signaling pathway, Jak-STAT signaling pathway, inflammatory mediator regulation of TRP channels and AMPK signaling pathway. Among the 26 pathways, 16 pathways were also enriched by 24 targets shared by Salvia miltiorrhizae and Panax notoginseng, including the TNF signaling pathway, VEGF signaling pathway, apoptosis, HIF-1 signaling pathway, toll-like receptor signaling pathway, neurotrophin signaling pathway, sphingolipid signaling pathway, insulin signaling pathway, PI3K-Akt signaling pathway, prolactin signaling pathway, MAPK signaling pathway, estrogen signaling pathway, cholinergic synapse, serotonergic synapse, mTOR signaling pathway and ErbB signaling pathway. These 16 pathways may constitute the same major functions of Salvia miltiorrhizae and Panax notoginseng in the treatment of pain. Inhibition of tumor necrosis factor-alpha (TNF-α) can reduce inflammation and pain^28^. Toll-like receptors are now recognized to contribute to the chronic pain process^29^. Moreover, myocyte apoptosis was detected as a possible promoter of pain and motor dysfunction in neuropathic rats^30^. These 26 pathways can be classified into the following categories: environmental information processing, organismal systems, metabolism and cellular processes. Pathways in signal transduction (11) account for the most pathways. Pain is transmitted by neurons and nerve conduction. The nerve conduction of pain can be classified into four cascades, including pain sensing of the nociceptive receptor, pain transmission of the primary afferent fiber, dorsal horn of the spinal cord, spinal cord-fasciculus thalamicus and other ascending tracts, including pain integration in the cortical and limbic systems, descending control and pain modulation of neurotransmitters. Therefore, there is no doubt that many pathways in signal transduction are involved in pain signal transduction, and these pathways represent therapeutic targets in the treatment of pain. Pathways in the endocrine system (6) account for the second highest number of pathways. Many painful conditions appear to be induced, reduced, and in some cases, modulated by hormones; additionally, knowledge of the role of the endocrine system in chronic pain mechanisms is slowly increasing in experimental and clinical studies^31^. Pathways in the nervous system (3) and lipid metabolism (3) account for the third highest number of pathways. Pain is a nervous system disease. Specialized pro-resolving lipid mediators have a function in pain^32^. Analgesic lipid mediators include enzyme pathways, such as endogenous agonists of cannabinoid receptors (endocannabinoids), lipid-amide agonists of peroxisome proliferator-activated receptor-α, and products of oxidative metabolism of polyunsaturated fatty acids via cytochrome P450. These lipid messengers are produced and act at different stages of the response to tissue injury and may be part of a peripheral gating mechanism that regulates the access of nociceptive information to the spinal cord and brain^33^.

**Table 3:** Fourteen pathways related to pain are significantly enriched by 223 targets of FFDS.

KEGG pathway	Count	P-Value	Fold Enrichment	Bonferroni	Class
TNF signaling pathway	23	2.56E-14	8.15	6.13E-12	Environmental information processing; signal transduction
Arachidonic acid metabolism	16	3.91E-11	9.69	9.34E-09	Metabolism; lipid metabolism
HIF-1 signaling pathway	18	5.19E-10	6.90	1.24E-07	Environmental information processing; signal transduction
Toll-like receptor signaling pathway	18	1.84E-09	6.38	4.40E-07	Organismal systems; immune system
Adipocytokine signaling pathway	15	2.68E-09	8.05	6.42E-07	Organismal systems; endocrine system
Linoleic acid metabolism	10	3.34E-08	12.95	7.98E-06	Metabolism; lipid metabolism
Serotonergic synapse	16	1.83E-07	5.41	4.38E-05	Organismal systems; nervous system
PI3K-Akt signaling pathway	28	3.12E-07	3.05	7.42E-05	Environmental information processing; signal transduction
Apoptosis	12	5.22E-07	7.27	1.25E-04	Cellular processes; cell growth and death
Steroid hormone biosynthesis	11	2.30E-06	7.12	5.49E-04	Metabolism; lipid metabolism
NF-kappa B signaling pathway	13	2.60E-06	5.61	6.21E-04	Environmental information processing; signal transduction
VEGF signaling pathway	11	3.71E-06	6.77	8.86E-04	Environmental information processing; signal transduction
mTOR signaling pathway	10	1.83E-05	6.47	4.36E-03	Environmental information processing; signal transduction
MAPK signaling pathway	20	4.03E-05	2.95	9.58E-03	Environmental information processing; signal transduction

### The relationships of herbs, compounds and FFDS targets with pain disease proteins

We built the FFDS targets-other human proteins PPI network and pain disease proteins-other human proteins PPI network. We used 141 pain disease proteins (Table S4) and 223 FFDS targets to identify FFDS-related pain disease proteins. Pain disease proteins that can be directly targeted by FFDS were treated as FFDS primary pain disease proteins. Pain disease proteins that can directly interact with FFDS targets but cannot be directly targeted by FFDS were treated as FFDS secondary pain disease proteins. FFDS primary and secondary pain disease proteins can be defined as FFDS-related pain disease proteins. Fifty-six FFDS-related pain disease proteins (Table S5) were identified, including 10 primary pain disease proteins and 46 secondary pain disease proteins. The primary pain disease proteins include catechol O-methyltransferase (COMT), endothelin-1 (EDN1), interleukin-2 (IL2), RAC-alpha serine/threonine-protein kinase (AKT1), neuronal acetylcholine receptor subunit alpha-7 (CHRNA7), prostaglandin G/H synthase 2 (PTGS2), serum albumin (ALB), protachykinin-1 (TAC1), oxytocin-neurophysin 1 (OXT) and TNF. Five primary pain disease proteins have direct interactions with the pain disease protein set, including AKT1, ALB, PTGS2, TNF and COMT, and the minimum path lengths are 2 between any of the other primary pain disease proteins and the pain disease protein set except for IL2 (3). Two proteins may have no direct interaction, but they may have indirect connections through other human proteins called intermediators, which form several paths to connect these proteins. The minimum path length can show whether a protein has close interaction or distant interaction with a protein set (without that protein). AKT1 and ALB can interact with most pain disease proteins. Among the 46 secondary pain disease proteins, 27 proteins have direct interactions with the pain disease protein set, and the minimum path lengths between any of the other 17 secondary pain disease proteins and the pain disease protein set (except for the proteins themselves) are 2, and the minimum path lengths are 3 and 4 for the remaining two secondary pain disease proteins. Among the 27 secondary pain disease proteins that have direct interactions with the pain disease protein set, high affinity nerve growth factor receptor (NTRK1) can interact with the most pain disease proteins (14) directly, followed by transitional endoplasmic reticulum ATPase (VCP) (11), heterogeneous nuclear ribonucleoprotein A1 (HNRNPA1) (9) and RNA-binding protein FUS (FUS) (8). These results suggest that all 56 FFDS-related pain disease proteins have direct or indirect connections with the pain disease protein set except for themselves.

**Figure 1:**
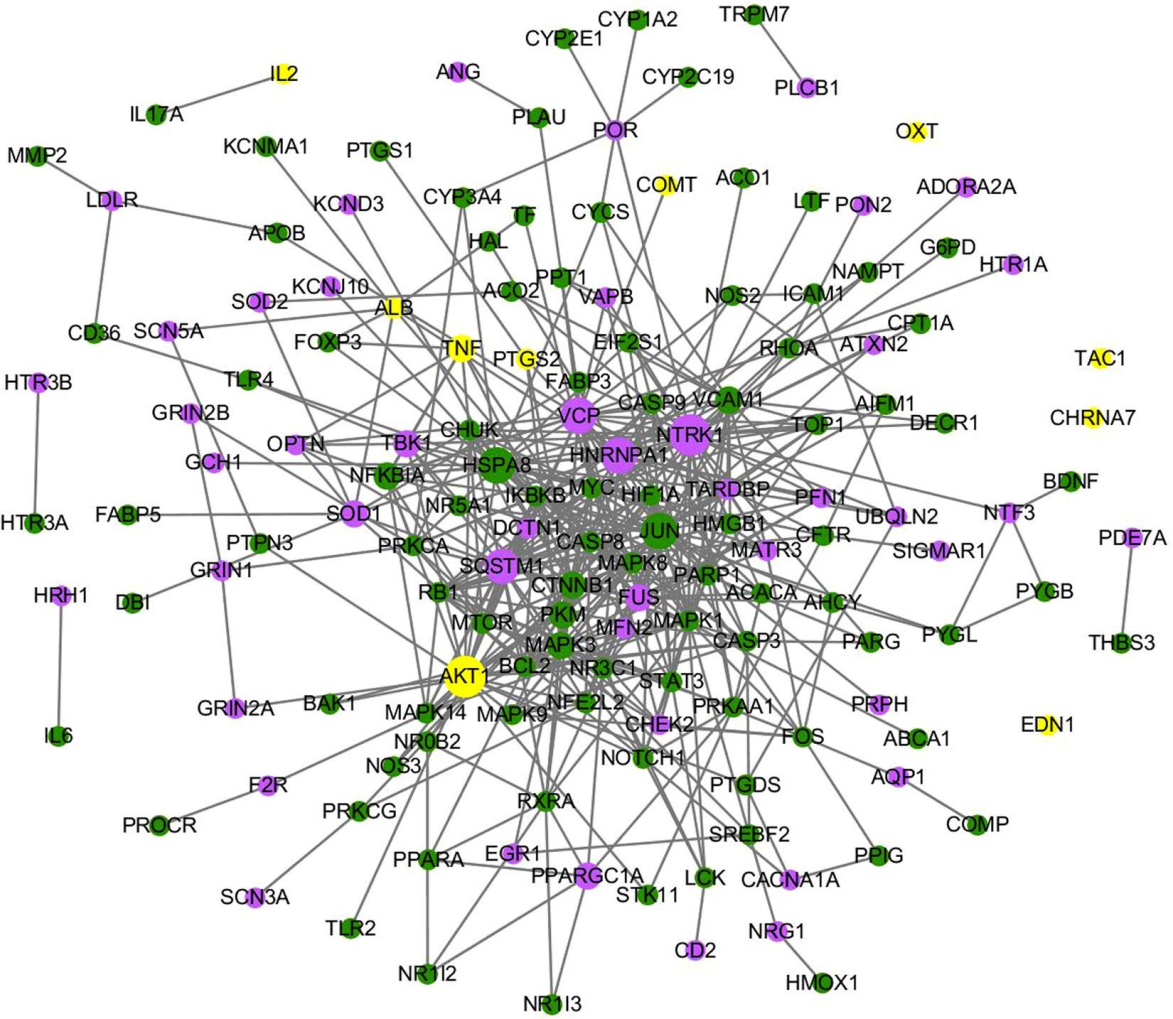
The network for 56 FFDS-related pain disease proteins and their interactive FFDS targets. Green nodes represent FFDS targets, blue nodes represent pain disease proteins, and yellow nodes represent FFDS targets/pain disease proteins.

Six primary pain disease proteins belong to Panax notoginseng, 3 belong to Salvia miltiorrhizae, and AKT1 is shared by Panax notoginseng and Salvia miltiorrhizae. AKT1 can be targeted by three compounds, while COMT, EDN1 and IL2 can be targeted by two compounds in Salvia miltiorrhizae. PTGS2 can be targeted by two compounds in Panax notoginseng, and CHRNA7, ALB, TAC1, OXT and TNF can be targeted by one compound in Panax notoginseng. AKT1 is involved in the PI3K-Akt signaling pathway. IL2 is a cytokine; cytokines play an important role in pain through different mechanisms in several sites of pain transmission pathways^34^. PTGS2 inhibitors have been widely used to treat inflammation and pain^35,36^. As shown in Fig. 1, we built a network for the 56 FFDS-related pain disease proteins and their interactive FFDS targets. AKT1 is a FFDS primary pain disease protein, and it interacts with the most FFDS targets (19), followed by TNF (5). Among the 46 secondary pain disease proteins, NTRK1 is associated with most FFDS targets (23), followed by VCP (19), HNRNPA1 (16) and sequestosome-1 (SQSTM1) (14). NTRK1 and VCP can be indirectly targeted by 13 compounds in the three herbs, HNRNPA1 can be indirectly targeted by 12 compounds in three herbs, and SQSTM1 can be targeted by 11 compounds in three herbs; all of these proteins are associated with the highest number of compounds. Nerve growth factor (NGF) is a neurotrophic factor that acts as a mediator of pain, and NTRK1 encodes a receptor tyrosine kinase for NGF^37^. In addition, heat shock cognate 71 kDa protein (HSPA8) and transcription factor AP-1 (JUN) are FFDS targets instead of pain disease proteins, but they are associated with most pain disease proteins. Thus, HSPA8 and AP-1 may be factors that contribute to the therapeutic effects of FFDS in the treatment of pain. HSPA8 is implicated in a wide variety of cellular processes, including the protection of the proteome from stress, folding and transport of newly synthesized polypeptides, activation of proteolysis of misfolded proteins and the formation and dissociation of protein complexes. JUN directly interacts with specific target DNA sequences to regulate gene expression.

Salvia miltiorrhizae possesses 36 pain disease proteins, and four of these proteins can be directly targeted by it. Panax notoginseng possesses 49 pain disease proteins, and seven of these proteins can be directly targeted by it. Borneolum possesses 12 pain disease proteins, and none of them can be directly targeted by it. Twenty-eight compounds in FFDS have direct or indirect interactions with pain disease proteins. Eicosatetraenoic acid in Salvia miltiorrhizae can directly target the most pain disease proteins (5) and is directly or indirectly related to the most pain disease proteins (29). Polysaccharide, ursolic acid, hexadecanoic acid, baicalin, rosmarinic acid and oleanolic acid are directly or indirectly related to at least ten pain disease proteins. We retrieved the literature in PubMed and found that 13 compounds in FFDS have antinociceptive effects. These compounds include ursolic acid, baicalin, rosmarinic acid, ferulic acid, caffeic acid, tanshinone IIA, protocatechuic acid, tetramethylpyrazine and gamma-sitosterol in Salvia miltiorrhizae; polysaccharide, ginsenoside Rg1 and ginsenoside Rg3 in Panax notoginseng; and oleanolic acid in Borneolum. For example, ursolic acid has anti-inflammatory, antioxidant, and antinociceptive effects in different animal models^38^. Polysaccharide pretreatment significantly reduced the number of writhes and licking time but did not increase the latency time of responses, demonstrating its antinociceptive effects^39,40^. Orally administered oleanolic acid showed an antinociceptive effect in a dose-dependent manner as measured in the acetic acid-induced writhing test^41^. We used a scoring system to assess herbs and compounds in FFDS that affect pain disease proteins and found that 13 compounds (ursolic acid:3.65, polysaccharide:3.13, baicalin:2.17, oleanolic acid:1.78, rosmarinic acid:1.33, ferulic acid:1.08, caffeic acid:1.04, tanshinone IIA:0.92, protocatechuic acid:0.76, tetramethylpyrazine:0.73, gamma-Sitosterol:0.52, ginsenoside Rg3:0.28 and ginsenoside Rg1:0.22. P <= 0.01) with known antinociceptive effects had scores. Additionally, 15 other compounds (eicosatetraenoic acid:4.90, hexadecanoic acid:3.21, ginsenoside Rd:1.96, ginsenoside Rg2:1.60, tanshinone I:1.07, ginsenoside Re:0.79, pentadecanoic acid: 0.71, cryptotanshinone:0.63, magnesium lithospermate B:0.44, ginsenoside Rh1:0.29, dauricine:0.17, miltirone:0.17, isoferulic acid:0.16, Danshensu:0.12 and asiatic acid:0.03; P <= 0.01 except for Dauricine and miltirone (P = 0.029)) in FFDS had scores. The maximum score, mean score and minimum score of the 13 known compounds are 3.65, 1.36 and 0.22, respectively, and the maximum score, mean score and minimum score of the 15 unknown compounds are 4.90, 1.08 and 0.03, respectively. We compared unknown compounds with known compounds by using t-tests and found no significant difference in the score values between them (P = 0.57), indicating that these unknown compounds are of equal importance in the treatment of pain. Six known compounds could directly target pain disease proteins. At least two targets (including pain disease proteins) of each of the 13 known compounds have direct associations with pain disease proteins. The percentages (number of targets directly associated with pain disease proteins (including pain disease proteins)/all targets of a compound) of 10 known compounds exceed 0.5. Additionally, the scores of 11 known compounds exceed 0.5. To improve prediction accuracy, we required satisfying at least two of the following conditions: directly targeting a pain disease protein, at least 2 targets with direct associations with pain disease proteins (including pain disease proteins), percentage >= 0.5 and score >= 0.5 to determine that a compound has effects in the treatment of pain. Indeed, all known compounds can be determined to have effects in treating pain by using this method. This method has been proven reliable in screening active compounds from herbal formulas. Using this method, eicosatetraenoic acid, hexadecanoic acid, ginsenoside Rd, ginsenoside Rg2, tanshinone I, ginsenoside Re, pentadecanoic acid, cryptotanshinone, magnesium lithospermate B, isoferulic acid, danshensu and asiatic acid can be determined to have effects in treating pain. These compounds and the 13 known compounds may be the main active ingredients of FFDS in treating pain. Relevant information about these compounds and the 13 known compounds is listed in Table 4. However, scores always show the comprehensive relevance of compounds to pain. The scores of eicosatetraenoic acid, hexadecanoic acid, ginsenoside Rd, ginsenoside Rg2, tanshinone I, ginsenoside Re and pentadecanoic acid in the 15 unknown compounds exceed 0.7, and these compounds have more potential as drugs for pain. In fact, the scores of 7/15 unknown compounds exceed 0.7, while the scores of 10/13 known compounds exceed 0.7.

**Table 4:** Relevance information about herbs and compounds (determined to have effects in treating pain) with regard to pain.

Herb/compound	n	k	Number of directly targeted pain disease proteins	Percentage (k/n)	Sum score	Max score	P-Value	Type
Salvia miltiorrhizae (herb)	99	58	4	0.586	14.97	0.54	0.00	known
Ursolic acid	16	13	1	0.812	3.65	0.54	0.00	known
Baicalin	23	13	0	0.565	2.17	0.42	9.55E-15	known
Rosmarinic acid	7	6	1	0.857	1.33	0.37	4.23E-09	known
Ferulic acid	16	7	0	0.438	1.08	0.36	1.64E-07	known
Tanshinone I	7	5	0	0.714	1.07	0.45	4.25E-07	unknown
Caffeic acid	20	5	1	0.250	1.04	0.40	2.28E-04	known
Tanshinone IIA	11	8	1	0.727	0.93	0.22	8.05E-11	known
Protocatechuic acid	4	3	1	0.750	0.76	0.43	9.75E-05	known
Tetramethylpyrazine	3	2	0	0.667	0.73	0.49	2.51E-3	known
Cryptotanshinone	4	3	1	0.750	0.63	0.35	9.75E-05	unknown
Gamma-Sitosterol	4	4	0	1.000	0.52	0.15	7.28E-07	known
Magnesium lithospermate B	5	3	1	0.600	0.44	0.19	2.39E-04	unknown
Isoferulic acid	3	2	0	0.667	0.16	0.12	2.51E-03	unknown
Danshensu	2	2	0	1.000	0.12	0.06	8.54E-04	unknown
Panax notoginseng (herb)	143	43	7	0.301	17.78	0.67	0.00	known
Eicosanetetraenoic acid	39	16	5	0.410	4.90	0.67	5.00E-15	unknown
Hexadecanoic acid	48	15	2	0.313	3.21	0.63	4.15E-12	unknown
Polysaccharide	14	14	0	1.000	3.13	0.53	0.00	known
Ginsenoside Rd	3	3	1	1.000	1.96	0.62	2.49E-05	unknown
Ginsenoside Rg2	3	3	0	1.000	1.60	0.36	2.49E-05	unknown
Ginsenoside Re	4	4	0	1.000	0.79	0.39	7.28E-07	unknown
Pentadecanoic acid	2	2	0	1.000	0.71	0.33	8.54E-04	unknown
Gamma-Sitosterol	4	4	0	1.000	0.52	0.15	7.28E-07	known
Ginsenoside Rg3	4	3	0	0.750	0.28	0.13	9.75E-05	known
Ginsenoside Rg1	5	2	1	0.400	0.22	0.14	8.05E-03	known
Dauricine	1	1	0	1.000	0.17	0.17	2.92E-02	unknown
Borneolum (herb)	17	11	0	0.647	1.80	0.37	1.38E-13	known
Oleanolic acid	14	9	0	0.643	1.78	0.37	2.70E-11	known
Asiatic acid	3	2	0	0.667	0.03	0.03	2.51E-03	unknown

Lines 2, 17 and 29 are herbal items. Compounds below an herb belong to this herb. The “Materials and methods” section contains definitions for n and k.

### Overlap analysis between FFDS targets and drug targets with indications for pain

To explore similarities between FFDS and known pain drugs, we collected known drugs in treating pain as well as their affected targets in humans from the DrugBank and TTD databases. These drug targets can be used as therapeutic targets. Subsequently, we built a drug-target network to explore frequently affected therapeutic targets. If a therapeutic target is affected by more drugs in treating the same disease, this target has more potential as an efficient approach for treating the disease. After we analyzed the networks (Table S6), the Mu-type opioid receptor (OPRM1) was targeted by the most drugs (55), and PTGS2 was targeted by the most approved drugs (14), indicating that they are efficient therapeutic approaches for pain treatment. PTGS2 can be directly targeted by FFDS. Prostaglandin G/H synthase 1 (PTGS1) is associated with 22 drugs, including 8 approved drugs, and can be directly targeted by FFDS.

**Figure 2:**
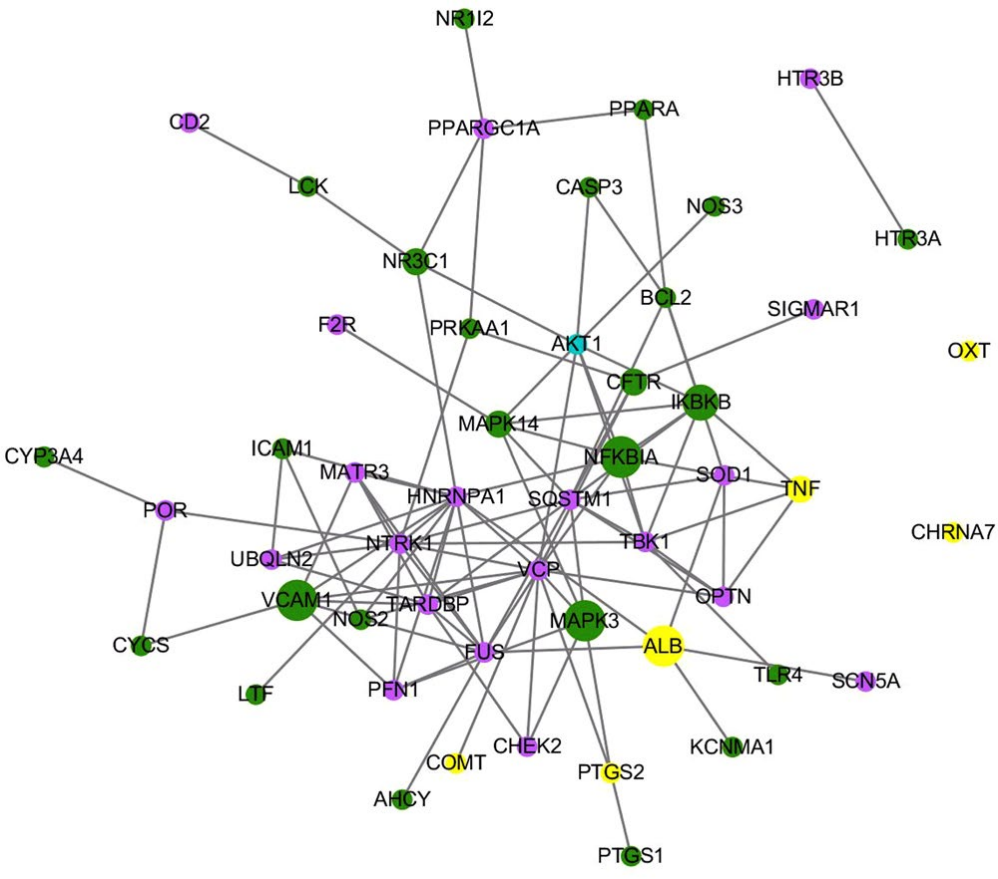
The direct interactions between 45 common therapeutic targets and pain disease proteins. Green nodes represent therapeutic targets, blue nodes represent pain disease proteins, and yellow nodes represent therapeutic targets/pain disease proteins.

In total, 45 therapeutic targets are affected by both FFDS and known drugs. All therapeutic targets have direct or indirect connections with the pain disease protein set. Six therapeutic targets are also pain disease proteins, including PTGS2, CHRNA7, COMT, TNF, ALB and OXT, while 24 therapeutic targets are not pain disease proteins but can directly interact with the pain disease proteins. For the remaining 15 therapeutic targets, the minimum path lengths between each target and pain disease proteins are 2. As shown in Fig. 2, we built a network to show the direct connections between the 45 therapeutic targets and pain disease proteins. For the six therapeutic targets/pain disease proteins, ALB can interact with 4 other pain disease proteins, TNF can interact with 2 other pain disease proteins, and PTGS2 and COMT can interact with 1 other pain disease protein. By contrast, CHRNA7 and OXT have no direct interactions with other pain disease proteins. Vascular cell adhesion protein 1 (VCAM1), which is not a pain disease protein, can directly interact with the most pain disease proteins (6), followed by NF-kappa B inhibitor alpha (NFKBIA) (5), mitogen-activated protein kinase 3 (MAPK3) (5) and inhibitor of nuclear factor kappa B kinase subunit beta (IKBKB) (4). Seven therapeutic targets associated with at least four known drugs are listed in Table 5. According to the enrichment results, the 45 therapeutic targets were significantly associated with 13 pain-related pathways, including the TNF signaling pathway, NF-kappa B signaling pathway, apoptosis, HIF-1 signaling pathway, Toll-like receptor signaling pathway, serotonergic synapse, VEGF signaling pathway, arachidonic acid metabolism, adipocytokine signaling pathway, cholinergic synapse, mTOR signaling pathway, sphingolipid signaling pathway and neurotrophin signaling pathway. All of these pathways could be found in the 26 pathways enriched by all FFDS targets. When we compared those pathways enriched by the 45 therapeutic targets and pathways enriched by all FFDS targets, we found that some of the 45 therapeutic targets and other FFDS targets were involved in the same pathways.

**Table 5:** The 7 common targets associated with the most drugs known for the treatment of pain.

Gene	Protein	Compound	Type
PTGS2	Prostaglandin G/H synthase 2	Hexadecanoic acid, eicosatetraenoic acid	successful
PTGS1	Prostaglandin G/H synthase 1	Eicosatetraenoic acid	successful
HTR3A	5-hydroxytryptamine receptor 3A	Ginsenoside Rg3	successful
NR3C1	Glucocorticoid receptor	Ginsenoside Re	investigational
CHRNA7	Neuronal acetylcholine receptor subunit alpha-7	Ginsenoside Rg1	investigational
TOP2A	DNA topoisomerase 2-alpha	Ursolic acid, oleanolic acid	investigational
ALOX5	Arachidonate 5-lipoxygenase	Baicalin, caffeic acid, eicosatetraenoic acid	investigational

### Evaluation of FFDS targets and analysis of significant targets

**Figure 3:**
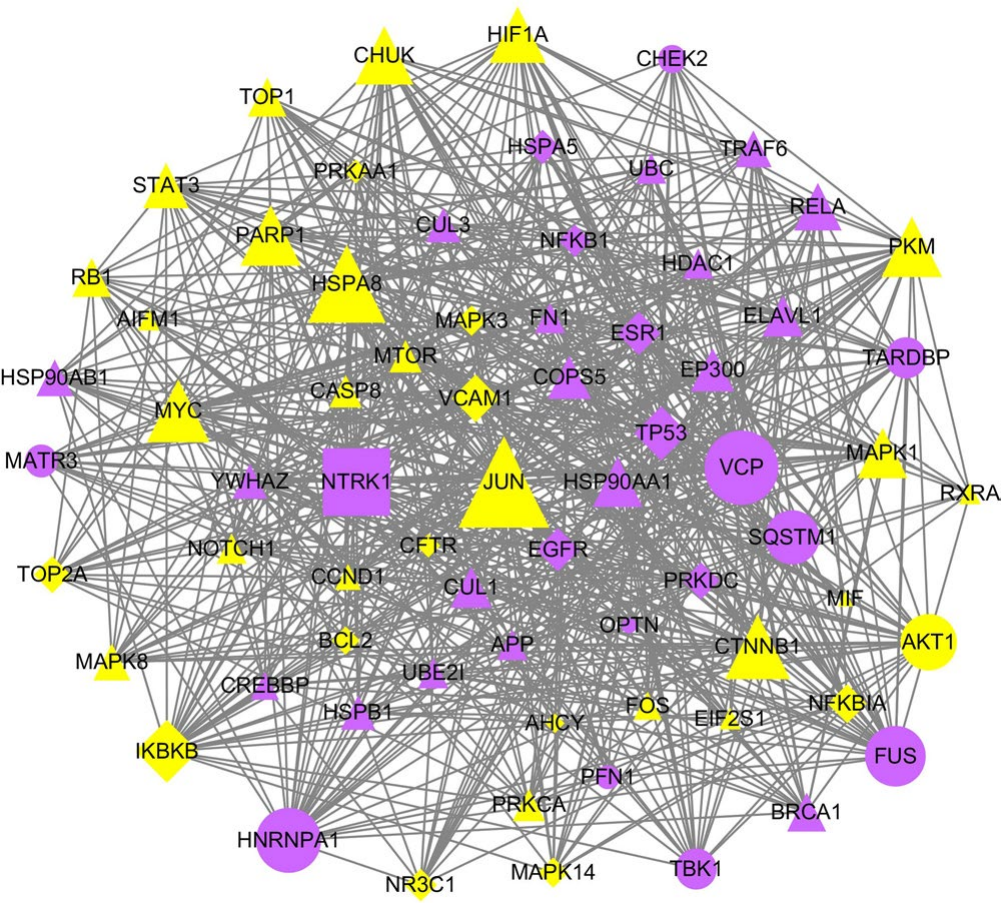
A highly connected subnetwork in the FFDS targets-pain disease proteins-other human proteins network. Triangle nodes correspond to FFDS targets or other human proteins, circle nodes are pain disease proteins, diamond nodes are therapeutic targets, rectangle nodes represent therapeutic targets/pain disease proteins, yellow nodes can be directly targeted by FFDS, and blue nodes can be indirectly affected by FFDS.

Highly connected proteins in PPIs can be defined as significant proteins. To identify the significant targets of FFDS, we constructed a FFDS targets-pain disease proteins-other human proteins PPI network to evaluate FFDS targets. We calculated the degrees of FFDS targets, and 109 FFDS targets (Table S7) with higher degrees than the median value of the degrees of all FFDS targets in the network can be defined as significant targets of FFDS. The degrees of JUN, Myc proto-oncogene protein (MYC), VCAM1, HSPA8, Catenin beta-1 (CTNNB1) and AKT1 are the highest at 1474, 892, 656, 428, 368 and 314, respectively. VCAM1 and AKT1 are therapeutic targets of drugs with indications for pain and pain disease protein, respectively. Five significant targets (AKT1, ALB, TNF, COMT, and PTGS2) are pain disease proteins, and ALB, TNF, COMT and PTGS2 are also therapeutic targets. There are 25 therapeutic targets except for those four therapeutic targets/pain disease proteins. As shown in Fig. 3, a highly connected subnetwork was extracted from the FFDS targets-pain disease proteins-other human proteins PPI network. The highly connected subnetwork can be defined as a core of FFDS. The highly connected subnetwork includes 29 pain disease proteins or therapeutic targets of drugs with indications for pain. Thus, FFDS may significantly affect pain through this network.

Then, we performed pathway enrichment analysis, and 109 significant targets were significantly associated with 28 pathways related to pain. The TNF signaling pathway, NF-kappa B signaling pathway, apoptosis, HIF-1 signaling pathway, Toll-like receptor signaling pathway, serotonergic synapse, VEGF signaling pathway, adipocytokine signaling pathway, cholinergic synapse, mTOR signaling pathway, sphingolipid signaling pathway and neurotrophin signaling pathway were also enriched by all FFDS targets and 45 therapeutic targets. This finding indicates that the significant therapeutic targets are involved in those pathways. The PI3K-Akt signaling pathway, MAPK signaling pathway, ErbB signaling pathway, prolactin signaling pathway, oxytocin signaling pathway, insulin signaling pathway, GnRH signaling pathway, estrogen signaling pathway, AMPK signaling pathway and inflammatory mediator regulation of TRP channels were also enriched by all FFDS targets. Other pathways, including the cAMP signaling pathway, retrograde endocannabinoid signaling, Wnt signaling pathway, dopaminergic synapse and Rap1 signaling pathway, can be classified into environmental information processing (signal transduction) and organismal systems (nervous system). VEGF is a selective endothelial cell mitogen that promotes angiogenesis and increases blood vessel permeability. Inhibiting VEGF provides effective pain relief^42^. Endocannabinoids modulate neuronal, glial and endothelial cell function and have neuromodulatory, anti-excitotoxic, anti-inflammatory and vasodilatory effects. Endocannabinoids behave as analgesics in acute nociception and clinical pain models, such as inflammation and painful neuropathy^43,44^.

## Discussion

Pain is a severe symptom related to many diseases as well as a disease itself that affects thousands of people. As quality of life has improved and the need for painless treatment is increasing, studies on pain are attracting more attention than ever. Though many new analgesics have been introduced to the clinic, pain remains an issue. Additionally, alternative analgesics are required because of the side effects of currently available analgesics. Chinese herbal formulas are well known for their advantages in curing complex diseases with few side effects. FFDS is a Chinese herbal formula that has significant therapeutic effects on attenuating pain. FFDS has been widely used to treat various diseases. One common symptom among these diseases, such Alzheimer’s disease, is pain; thus, determining the mechanisms of FFDS in attenuating pain will aid in the development of many new therapeutic approaches for patients in pain and provide better understanding of pain in the treatment of other diseases.

In the present study, we conducted a bioinformatics investigation to uncover the potential pharmacological mechanisms of FFDS in the treatment of pain. A large-scale text mining revealed that three herbs in FFDS have effects on pain, and some active compounds in each herb have antinociceptive effects. New drugs that may have analgesic effects were discovered based on drug-target principles, including tanshinone I in Salvia miltiorrhizae, eicosatetraenoic acid, hexadecanoic acid, ginsenoside Rg2 and ginsenoside Rd in Panax notoginseng. The pathways constituting the foundational mechanisms of the effects of FFDS on pain have been enriched by 223 targets of FFDS, and they can be classified into signal transduction, endocrine system, lipid metabolism and nervous system. Salvia miltiorrhizae and Panax notoginseng can significantly affect different pathways related to pain, but they also contribute to the effects of the opposite herb in a corresponding pathway, such as the HIF-1 signaling pathway, PI3K-Akt signaling pathway, TNF signaling pathway and NF-kappa B signaling pathway.

Pain disease proteins and therapeutic targets of drugs with indications for pain are directly or indirectly affected by FFDS, and these proteins may be the main factors that FFDS targets to treat pain. Most pain disease proteins and therapeutic targets are highly connected in the PPI network and can be defined as significant proteins. By comparing their locations in KEGG pathways, we found that most of these proteins were located at the beginning of these pathways or at important points within the pathways. Targeting these proteins can affect an entire pathway and has significant therapeutic effects. Other targets of FFDS can affect many pain disease proteins, which cannot be ignored, such as HSPA8 and JUN. Of course, a single drug affects only a few therapeutic targets. However, a formula can affect more targets due to its complex composition. Except for those disease proteins and therapeutic targets, other targets of FFDS were also involved in the same pathways as those disease proteins and therapeutic targets. These “unnecessary targets” were located in unimportant locations or could be indirectly affected by the disease proteins and therapeutic targets. “Unnecessary targets” may bridge several points to enhance the functions of FFDS or they may be therapeutic targets that have not yet been discovered. Moreover, we speculate that for a pathway related to a disease, affecting only a small part of the pathway is necessary to cure this disease; thus, these “unnecessary targets” are affected by FFDS only to reverse its side effects on the human body. Some “unnecessary targets” may be harmful to the human body; therefore, the formula should be optimized.

Based on the published literature, we can conclude that FFDS has therapeutic effects in the treatment of neuropathic pain, cancer pain and arthralgia. In terms of neuropathic pain, three disease proteins of neuropathic pain can be directly targeted by FFDS, including TNF^45^, PTGS1^46^ and PTGS2^47^; twenty therapeutic targets of drugs with indications including neuropathic pain can be directly affected by FFDS, such as PTGS2, PTGS1 and 5-hydroxytryptamine receptor 3A (HTR3A), which have all been associated with at least four drugs known for the treatment of neuropathic pain. Additionally, fourteen pathways that have relationships with neuropathic pain therapy can be significantly enriched by targets of FFDS, for example, the TNF signaling pathway^45^, Toll-like receptor signaling pathway^48^, PI3K-Akt signaling pathway^49^ and apoptosis^50^. No disease proteins for cancer pain can be directly targeted by FFDS, while one therapeutic target (HTR3A) can be directly targeted by FFDS, and two pathways (HIF-1 signaling pathway^51^ and T cell receptor signaling pathway)^52^ that have relationships with cancer pain therapy are significantly enriched by FFDS targets. FFDS has proven anticancer properties; in our investigation, many cancer-related proteins and pathways could be affected by FFDS. Thus, many indirect factors should be affected by FFDS to attenuate cancer pain, including proteins that can interact with cancer pain-related proteins. Seven disease proteins for arthralgia can be directly targeted by FFDS, including macrophage migration inhibitory factor (MIF), interleukin-6 (IL6), 72 kDa type IV collagenase (MMP2), Toll-like receptor 4 (TLR4), connective tissue growth factor (CTGF), cartilage oligomeric matrix protein (COMP) and interleukin-10 (IL10). Arthrogenic alphaviruses can cause debilitating illnesses characterized by arthritis and arthralgia, and evidence suggests that both MIF and CD74 play a critical role in mediating alphaviral disease^53^. In the chronic phase, the level of IL-6 has been associated with persistent arthralgia, providing a possible explanation for the etiology of arthralgia that plagues CHIKV-infected patients^54^. No therapeutic targets of drugs with indications including arthralgia can be targeted by FFDS. Two pathways (the Toll-like receptor signaling pathway^55^ and osteoclast differentiation)^56^ that have relationships with arthralgia can be significantly enriched by FFDS targets. Based on our results, we can conclude that FFDS can treat the aforementioned diseases through a target network, not simply one or two targets. Due to space limitations, we cannot discuss many mechanisms of action of FFDS in the treatment of these diseases. Therefore, we suggest that more investigators should use TCM network pharmacology approaches to unveil these mechanisms. In conclusion, this paper has fully elucidated the potential mechanisms of the effects of FFDS on pain from a systemic perspective based on targets of FFDS and network analysis. Moreover, this study will provide basic theories for the usage of this formula and its herbs.

## Materials and Methods

### FFDS, its herbs and pain

We referred to an article and used a computational method to assess the relevance between FFDS, its herbs and pain. The more papers relating an herb to pain, the more efficient this herb will be in treating pain. A parameter was used to balance bias and further assess the relevance between FFDS, its herbs and pain. The parameter is the ratio of pain herb-related papers and disease herb-related papers. The P-value was calculated by Eq. (1) and used to examine the error.

1 $$P=1-\sum _{{\rm{i}}=0}^{{\rm{k}}-1}\frac{(\begin{array}{c}K\\ {\rm{i}}\end{array})(\begin{array}{c}N-K\\ n-i\end{array})}{(\begin{array}{c}N\\ n\end{array})}$$

where N is the total number of papers in PubMed (27 million), K is the number of papers related to pain (599207, using “Pain” as a keyword), n is the number of papers about FFDS or its herbs and diseases (using synonyms for FFDS or its herbs and disease as keywords), and k is the number of papers about FFDS or its herbs and pain (using synonyms for FFDS or its herbs and pain as keywords). We used synonyms of an item (such as Salvia miltiorrhizae, its synonyms are Salvia miltiorrhizae and Danshen, which are wildly used in papers) to search PubMed and download Pubmed IDs, then we removed duplicate IDs and calculated the total number of the searched papers.

### Data preparation

Herbal compounds in FFDS were collected and extracted from TCM database@TW^57^ (http://tcm.cmu.edu.tw/zh-tw/index.php, updated 2013 July) and PubMed literature. Therapeutic targets and their associated drugs were collected from DrugBank^58^ (https://www.drugbank.ca/, version 5.0) and Therapeutic Target Database^59^ (http://bidd.nus.edu.sg/BIDD-Databases/TTD/TTD.asp, last updated on Sep 10, 2015). Pain disease proteins were collected from the Therapeutic Target Database and DisGeNET^60^ (with score >= 0.2, http://www.disgenet.org, version 5.0). We retained only the drugs (their therapeutic targets) and disease proteins that clearly belonged to “Pain”. PPI data were obtained from Mentha^61^ (http://mentha.uniroma2.it/). The known targets of compounds in FFDS were collected from STITCH^62^ (http://stitch.embl.de/, version 5.0) with a higher interaction score (confidence >= 0.7).

### Drug-likeness and bioavailability analysis

Compounds with drug-likeness properties and high bioavailability are more likely to be drugs. To explore the drug-likeness and bioavailability of the compounds in FFDS, we used SwissADME (http://www.swissadme.ch/)^63,64^ as a tool to calculate the drug-likeness and bioavailability of each compound. The drug-likeness rules include Lipinski’s rule, Ghose’s rule, Veber’s rule, Egan’s rule and Muegge’s rule.

### Statistical analysis to assess the probabilities of compounds and herbs in FFDS in the treatment of pain

Uncovering active compounds in Chinese herbal formulas for specific diseases can facilitate the modernization of TCM. Here, we built a new computational method to simply assess compounds and herbs in FFDS in treating pain. First, we constructed the pain disease proteins-other human proteins PPI network to evaluate the significance of pain disease proteins for pain. Increased significance of a protein in the network indicates that this protein is more essential for pain^65^. According to the centrality of a protein in the PPI network, which has a strong correlation with the essentiality of a protein, the protein will be given a weight value Si to show its relative significance. First, we calculated the degrees of pain disease proteins in the pain disease proteins-other human proteins PPI network. Then, we calculated log10 values of degrees. Finally, we divided log10 values by the maximum log10 value for normalization. We used those normalized values as the weight values Si of each pain disease protein. As degrees 1 and 2 are similarly important and log10 of 1 is 0, we changed the weight values of pain disease proteins with degrees 1 or 2 to 0.4 (log10 of 3 is 0.4771) divided by the maximum log10 value. In truth, the degrees of only two pain disease proteins used here were 1 or 2. The probability scores of PPIs were obtained from the Mentha database. The product of the weight value of a pain disease protein and the maximum probability of a specified compound directly or indirectly interacting with that protein will be considered the computational relevance score of the compound with pain through this pain disease protein. The sum of the relevance scores of a compound with regard to all its directly or indirectly interactive pain disease proteins will be treated as the computational relevance score of the compound with pain through all its related pain disease proteins. Ultimately, the sum of relevance scores of all compounds in one herb was treated as the computational relevance score of the herb with pain through all its related pain disease proteins. The score could not represent the real therapeutic effects, but it may show the probabilities and relative significance of herbs and their compounds in the treatment of diseases. Similar to Eq. (1), we also used P-values to examine the possibility of finding that a certain number of proteins were targets of a compound or an herb and had direct associations with pain disease proteins (including pain disease proteins themselves) in at least k proteins by chance. Here, N is the total number of human proteins in UniProt (162191), and K is the number of proteins that have direct associations with pain disease proteins (including pain disease proteins) (4739). n is the number of targets of a compound or an herb, and k is the number of proteins that were targets of a compound or an herb and had direct associations with pain disease proteins (including pain disease proteins).

### Network construction and analysis

Many types of networks were built and analyzed to explore the pharmacological mechanisms of the effects of FFDS on pain. The degree was calculated to assess the significance of a protein in a network by NetworkAnalyzer in Cytoscape 3.2. The centrality-lethality rule shows that a highly connected protein is more important to an organism than a poorly connected protein^65,66^. All networks were visualized and analyzed by Cytoscape 3.2.

### Pathway enrichment and analysis

Database for Annotation, Visualization, and Integrated Discovery^67^ (DAVID, https://david.ncifcrf.gov/, version 6.8) was used as a tool for KEGG pathway enrichment by using default settings. Related pathways are available in the KEGG pathway database^68^ (KEGG: Kyoto Encyclopedia of Genes and Genomes, http://www.kegg.jp/, release 81.0).

## Supplementary information

Table S1

Table S2

Table S3

Table S4

Table S5

Table S6

Table S7
